# Functional and cosmetic advantages of gasless endoscopic thyroidectomy in papillary thyroid carcinoma: a randomized trial

**DOI:** 10.1186/s12957-025-04119-z

**Published:** 2025-12-29

**Authors:** Jianbo Li, Xiaoyan Yu, Chengping Lin, Gaofei He, Xiaoxiao Lu, Junjie Chu, Jinxi Jiang, Nizhen Xu, Qimin Zhang, Deguang Zhang

**Affiliations:** 1 Department of Head and Neck Surgery, Sir Run Run Shaw Hospital, School of Medicine, Zhejiang University, Hangzhou, China; 2 Second People’s Hospital of Linhai, Taizhou, Zhejiang PR China

**Keywords:** Subclavicular approach, Cosmetic, Functions of the anterior cervical region, PTC

## Abstract

**Background:**

Conventional open surgery (OP) is effective and simple for treating papillary thyroid cancer (PTC), but the excessive separation of band strap muscles causes discomfort in the anterior cervical region and a long scar, which may impair the patient’s life quality and beauty. Gasless endoscopic surgery via subclavicular approach (ESSA) may provide a better alternative.

**Methods:**

The prospective data of 90 PTC patients who underwent ESSA or OP (45:45) in hemithyroidectomy and central neck dissection (CND) in our center from March 2022 to August 2023 were analyzed. Safety indicator incidence of complications, efficiency indicator No. lymph nodes (LNs) of CND, postoperative recurrence of central neck LNs indicated by Ultrasound (US) at 6 months, postoperative functions of the anterior cervical region and cosmetic satisfaction were recorded as the primary endpoints.

**Results:**

The ESSA group had no significant differences with the OP group in baseline data, complications, No. LNs (9.04 ± 4.58 vs. 9.87 ± 4.89, *p* = 0.413) and metastatic LNs (1.60 ± 2.79 vs.1.69 ± 2.50, *p* = 0.874) of CND. The two groups had no postoperative recurrence of central neck LNs indicated by ultrasound (US) at 6 months. The ESSA group exhibited better functionally sensitive and motional outcomes in the anterior cervical region compared to the OP group (*P* = 0.0217 and *P* = 0.008), and had higher cosmetic satisfaction than the OP group (*P* < 0.001).

**Conclusion:**

Compared with OP, ESSA is equally safe and effective, but more cosmetic and conducive to preserving functions of the anterior cervical region. ESSA can be considered an alternative approach to OP in hemithyroidectomy and CND.

## Introduction

The incidence of thyroid cancer among young people has been increasing in many countries [1]. Surgery is the preferred curative method for most patients with well-differentiated thyroid cancer [2]. Conventional OP is performed via linea alba cervicalis approach, which leaves the neck a Kocher’s incision [3]. This widely used approach is minimally invasive, effective and safe. More importantly, it has a short learning curve and makes it easy to perform simultaneous bilateral surgery of the gland. However, this approach destroys the anterior cervical fat pad on the surface of strap muscles and separates the transcervical white line and the anterior and posterior fascia of strap muscles [4, 5], which will result in postoperative adhesions and finally lead to sensitive and motional discomfort in the anterior cervical region, and any discomfort and scar in the anterior cervical region reduce postoperative quality of life (QoL) [6–8].

Exploiting the natural gap between the sternal and the clavicle head of the sternocleidomastoid muscle (SCM) without opening the transcervical white line can effectively reduce postoperative adhesions and alleviate discomfort in the anterior cervical region. Moreover, concealed incisions can better meet the needs of young people who are threatened by the increasing incidence of thyroid cancer and improve their postoperative QoL. SCM intermuscular approaches mainly include axillary approach [9], supraclavicular approach [10] and subclavian approach [11]. Axillary approach is cosmetic, feasible and safe [12, 13], but its incision is so far away from the thyroid area that a longer distance is required between the subcutaneous fat and the pectoralis major muscle. The resulting more invasive operation, stricter surgical requirements and greater obstruction of the sternum and the clavicle make it more difficult to clean lymph nodes located anterior to the trachea. Supraclavicular approach offers the shortest distance from the thyroid gland, requires simple surgical techniques, and even allows the procedure to be performed under direct visualization. However, it cannot guarantee a completely invisible scar in the neck region and may result in suboptimal cosmetic outcomes. The results of a prospective cohort study show that supraclavicular approach is safe and effective in reducing postoperative symptoms and improving QoL [14]. The results of another clinical trial study on supraclavicular approach demonstrate that sternomastoid intramuscular approach is beneficial for preserving functions of the anterior cervical region, but the follow-up period in this study was too short to draw definitive conclusions [15]. The upper edge of the incision in subclavian approach is located within the dermatoglyphic folds of the subclavian edge, resulting in a scarless neck. Besides, most clothing can completely cover the incision scar, rendering its cosmesis far superior to that of supraclavicular approach. Furthermore, its incision is close to the thyroid area with a short learning curve [16], which can address the drawbacks associated with axillary approach, including excessive skin flap separation, excessive shielding of the sternum and the clavicle, and high surgical skill requirements. Subclavian approach combines the advantages of both supraclavicular and axillary approaches.

However, there are few clinical prospective studies on ESSA. Through comparing ESSA with traditional OP in unilateral thyroidectomy and CND from perspectives including safety, effectiveness, cosmesis and preservation of anterior cervical functions, we aimed to obtain evidence on the advantages of subclavian approach in persevering cosmetic outcomes and anterior cervical functions, in order to meet the needs of young thyroid cancer patients who are experiencing increasing incidence rate and to improve their QoL.

## Materials and methods

This randomized clinical trial followed the principles of the Declaration of Helsinki. Each of the patients included provided a written informed consent after fully understanding the trial. A copy of their written informed consents can be provided if needed. The protocol was approved by the Ethics Committee of Sir Run Run Shaw Hospital. This clinical trial was registered in National Medical Research Registration and Archival Information System (https://www.medicalresearch.org.cn, number: MR-33-22−001943).

### Patients

The patients who underwent unilateral lobectomy with CND in the Department of Head and Neck Surgery, Sir Run Run Shaw Hospital, School of Medicine, Zhejiang University from March 2022 to August 2023 were potential patients for this research. Other inclusion criteria: Preoperative fine-needle aspiration showed PTC with a tumor size ≤ 4 cm. Preoperative examination showed no evidence of external invasion or suspected LN metastasis in the lateral neck by US and computed tomography images. Exclusion criteria: patients aged < 18 years, with a prior history of thyroid surgery or severe underlying conditions, and with excessive obesity.

The surgeon elaborated the benefits and risks of ESSA and OP to potential patients and 101 patients expressed their willingness to participate in this trial and accept either approach. Subsequently, they were randomly divided into two groups (ESSA: OP = 50: 51).

### Surgery

OP: a 4–5 cm curved incision, made in the middle of the neck, approximately 1.5 cm above the sternal notch within the skin crease. The transcervical white line was separated to expose the thyroid gland lobes. The surgery was performed under direct visual inspection.

**Fig. 1:**
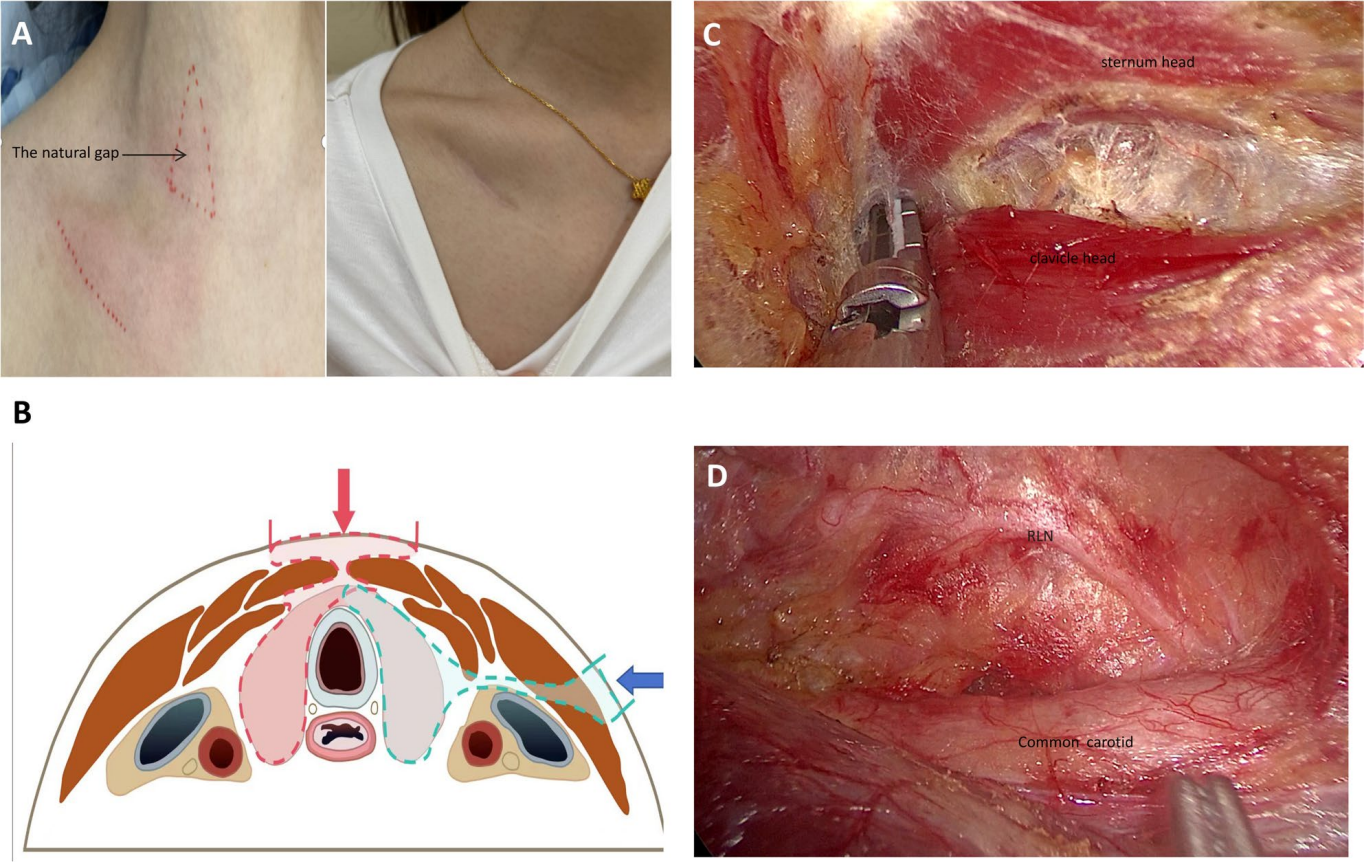
Incision and workspace of ESSA. **A** Incision and triangular space. **B** Surgical approach of two groups. **C** The natural gap between the SCM under endoscopic view. **D** Workspace of ESSA under endoscopic view. RLN: Recurrent laryngeal nerve. SCM: sternocleidomastoid muscle

ESSA: a 3.5 cm single oblique incision, whose upper edge was adjacent to the lower edge of the clavicle. The surgery was performed under laparoscopy through the natural gap between the posterior of the sternum head and the anterior of the clavicle head of the SCM. The separation range of the thyroid bed area was as follows: the upper boundary was at the level of the upper pole of the thyroid; the lower boundary was the suprasternal notch; the inner boundary was adjacent to the healthy side of the thyroid isthmus; the outer boundary was the carotid sheath. The surgeon should avoid excessive separation of the sternal thyroid muscle from the thyroid gland, and hook the thyroid gland with the sternal thyroid muscle to lift the thyroid gland inward and upward as a whole, so that it would be easier to dissect the recurrent laryngeal nerve and clean lymph nodes. When cleaning lymph nodes in the tracheoesophageal sulcus, attention should be paid to anatomical levels to avoid excessive depth and potential damage to the esophagus. The specific steps can refer to our previous reports (Fig. 1) [17].

### Clinical and pathological characteristics

Clinical characteristics including gender, age, location of cancer lesions, operation duration, bleeding volume, daily drainage volume (postoperative 48 h, ml), hospital stay (days), fluid accumulation, recurrent laryngeal nerve (RLN) injury and chyle leakage were extracted from Patient Database in the hospital. Pathological characteristics including T Stage, tumor size (largest diameter, cm), multifocality, thyroiditis, No. of LNs removed and No. of metastatic LNs were recorded in postoperative pathological reports.

Postoperative discomfort in the anterior cervical region (Sensitive and Motional) was recorded as a primary endpoint. It involved two evaluation items, each rated on a 0- to 4-point scale ranging from 0 = no symptoms to 4 = severe symptoms. The total score on anterior cervical function scale was the sum of item scores, with a lower score indicating fewer symptoms (Table 3). Meanwhile, cosmetic satisfaction was assessed as another primary endpoint and divided into four levels (highly satisfied, satisfied, neutral and unsatisfied). Satisfaction level was evaluated in a way similar to that for the evaluation of cosmetic satisfaction. Incision pain was assessed via visual analogue scale (VAS) from 0 to 10, with a lower score indicating less severe pain [18].

### Follow-up and quality control

Postoperative follow-up data were collected and recorded through telephone follow-up surveys and clinical communications by the same researcher at the designated time points after surgery.

All the surgeries were performed by experienced doctors from the same team. The researchers spared no effort to reduce bias. The recruited patients were divided into the two groups consecutively and randomly to decrease selection bias. In the mid-term of the research, the hospital and the provincial health commission implemented an inspection of this trial to ensure authenticity, randomness and blinding. Each patient was required to undergo an US examination by an experienced sonographer 6 months after surgery to evaluate tumor recurrence, for the purpose of achieving homogenization of the examination process.

### Statistical analysis

Statistical analysis was performed using Statistical Product and Service Solutions (SPSS) software version 26.0 (IBM Corp., Armonk, NY, USA). Continuous variables were expressed as mean ± standard deviation; unpaired sample t-test was used for comparison between groups; categorical variables were expressed as percentages (%); Pearson chi-square test was used to compare variables between groups. A difference with *p* < 0.05 was considered statistically significant.

## Results

**Fig. 2:**
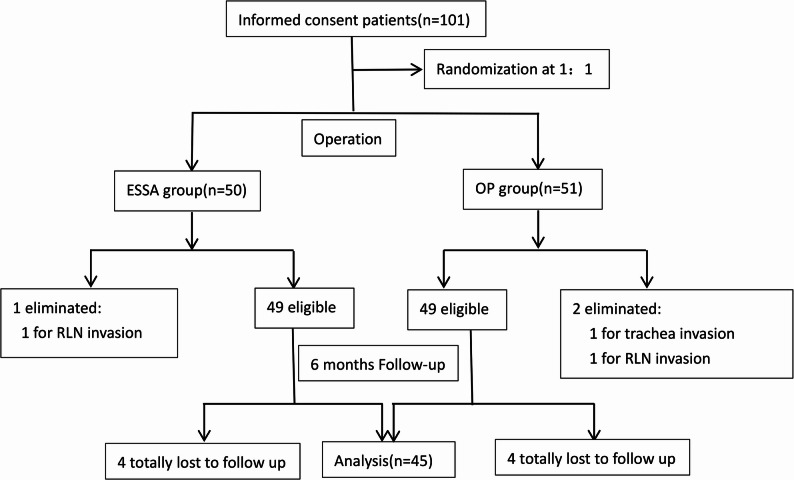
Study flow chart. RLN: recurrent laryngeal nerve

101 patients who underwent ESSA or OP in unilateral thyroidectomy and CND in our center from March 2022 to August 2023 were enrolled in the study. All the ESSA surgeries were completed successfully without conversion to OP. 1 patient from the ESSA group and 2 patients from the OP group were excluded due to RLN or trachea invasion. 4 patients from each group were lost to follow-up during the follow-up period. The flow chart of our study is shown in Fig. 2.

The clinical and pathological characteristics of the two groups are shown in Table 1, without significant differences. Most of the patients were female, with Male: Female being 10:35 vs. 13:32 (OP). The mean age of the ESSA group was slightly younger than lower than that of OP group, being 39.51 ± 10.25 years in the ESSA group and 42.64 ± 9.83 years in the OP group. The two groups were similar in terms of location of cancer lesions (Left: Right = 22:23). The T staging of most patients in both groups was T1a (≤ 1 cm), with only one patient in the ESSA group being T2 (>2 cm). The tumor size was 0.78 ± 0.51 in the ESSA group and 0.69 ± 0.28 in the OP group. Most tumors in the two groups were unifocal, while a small portion were multifocal, with a ratio of approximately 3:1. 19 patients in the ESSA group had thyroiditis and 14 patients in the OP group had thyroiditis. The No. of LNs removed in the central neck was 9.04 ± 4.58 in the ESSA group and 9.87 ± 4.89 in the OP group. The No. of metastatic LNs was 1.60 ± 2.79 in the ESSA group and 1.69 ± 2.50 in the OP group. They had no significant difference in No. of LNs as important indicator for evaluating effectiveness.

**Table 1:** Clinical and pathological characteristics of the ESSA and OP groups

	ESSA(*n* = 45)	OP(*n* = 45)	*p*-Value
Sex (M: F)	10:35	13:32	0.468
Age (years)	39.51 ± 10.25	42.64 ± 9.83	0.142
Location of cancer lesions			1.000
Left	22 (48.89)	22 (48.89)	
Right	23 (51.11)	23 (51.11)	
T stage^a^			0.538
cT1a	37 (82.22)	39 (86.67)	
cT1b	7 (15.56)	6 (13.33)	
cT2	1 (2.22)	0 (0.00)	
Tumor size (largest diameter, cm)	0.78 ± 0.51	0.69 ± 0.28	0.350
Multifocality			0.468
Absent	32 (71.11)	35 (77.78)	
Present	13 (28.89)	10 (22.22)	
Thyroiditis			0.274
Absent	26 (57.78)	31 (68.89)	
Present	19 (42.22)	14 (31.11)	
No. of LNs removed	9.04 ± 4.58	9.87 ± 4.89	0.413
No. of metastatic LNs	1.60 ± 2.79	1.69 ± 2.50	0.874

*LNs* Lymph nodes

^a^T Stage is according to Eighth Edition of the American Joint Committee on Cancer

### Surgical characteristics, postoperative hospitalization, complications

The two groups had no statistically significant differences in bleeding volume (17.00 ± 7.64 vs. 14.22 ± 5.74), daily drainage volume in postoperative 48 h (48.60 ± 17.65 vs. 42.13 ± 13.81), hospital stay (3.18 ± 0.68 vs. 2.91 ± 0.63) and parathyroid transplantation, while the ESSA group had longer operation duration than the OP group (119.93 ± 29.85 vs. 96.11 ± 28.36), with a statistically significant difference between the two groups. There were very few complications in both groups, with only one case of fluid accumulation in each group and no case of RLN injury and chyle leakage (Table 2).

**Table 2:** Surgical characteristics, Hospitalization, complications of ESSA and OP groups

	ESSA(*n* = 45)	OP(*n* = 45)	*p*-Value
Operative time (min)	119.93 ± 29.85	96.11 ± 28.36	< 0.001
Bleeding volume (ml)	17.00 ± 7.64	14.22 ± 5.74	0.054
Daily drainage volume ((postoperative 48 h, ml)	48.60 ± 17.65	42.13 ± 13.81	0.056
Hospital stay (days)	3.18 ± 0.68	2.91 ± 0.63	0.058
Fluid accumulation	1	1	1.000

none recurrent laryngeal nerve injury and chyle leakage

### Follow-up data

45 patients in each group were followed up for a follow-up period of 376.78 ± 131.97 vs. 367.20 ± 142.06 days. Their follow-up data were collected through telephone and clinical communications. All the patients were followed up for no less than 6 months. Pain was assessed via VAS in 24 h and one week after surgery. The OP group was more painful than the ESSA group with a statistically significant significance at postoperative 24 h, but they had no difference at postoperative 1 week. Regarding cosmetic satisfaction, the ESSA group was significantly higher than the OP group. When it came to satisfaction level, measured by satisfaction with the entire treatment process, the results of the two groups were the same, with 44 patients in each group expressing great satisfaction, 1 patient expressing satisfaction and no one expressing a neutral attitude or dissatisfaction. As an important observation indicator in this study, discomfort in the anterior cervical region was divided into two categories: sensitive discomfort and motional discomfort (Table 3). Follow-up was conducted at 1 week, 1 month, and 6 months after surgery. We found that there was no significant difference between the two groups in sensitive discomfort and motional discomfort at 1 week after surgery (*P* = 0.079, *P* = 0.617), However, there were statistically significant differences between the two groups in sensitive discomfort and motional discomfort at 1 month and 6 month after surgery (*P* = 0.004, *P* = 0.007 vs. *P* = 0.027, *P* = 0.008) (Table 4).

**Table 3:** Anterior cervical function score scale

Sensitive discomfort
Acupuncture discomfort at rest
A sense of neck tension at rest
Motional discomfort
Foreign body sensation when swallowing
Difficulty swallowing or discomfort when swallowing

Each item is rated on a 0- to 4-point from 0 = no symptoms to 4 = severe symptoms. The anterior cervical function score scale is the sum of item scores, lower scores indicate fewer symptoms

**Table 4:** Comparison of postoperative pain, cosmetic, level of satisfaction and anterior cervical region discomfort between ESSA and VAES groups

	ESSA(*n* = 45)	OP(*n* = 45)	*p*-Value
Follow-up time (months)	376.78 ± 131.97	367.20 ± 142.06	0.741
Postoperative pain (24 h)			
0	31 (68.89)	16 (35.56)	0.001
1	12 (26.67)	22 (48.89)	
2	2 (4.44)	7 (15.56)	
Postoperative pain (1 month)			
0	38 (84.44)	39 (86.67)	0.764
1	7 (15.56)	6 (13.33)	
Cosmetic satisfaction			< 0.001
Very satisfed	43 (95.56)	13 (28.89)	
Satisfed	2 (4.44)	25 (55.56)	
Average or Unsatisfed	0 (0.00)	7 (15.56)	
Level of satisfaction			1.000
Very satisfed	44 (97.78)	44 (97.78)	
Satisfed	1 (2.22)	1 (2.22)	
Average or Unsatisfed	0	0	
Sensitive discomfort (1 week)			0.079
0	43 (95.56)	38 (84.44)	
1	2 (4.44)	6 (13.33)	
2	0 (0.00)	1 (2.22)	
Sensitive discomfort (1 month)			0.004
0	44 (97.78)	35 (77.78)	
1	1 (2.22)	7 (15.56)	
2	0 (0.00)	3 (6.67)	
Sensitive discomfort (6 month)			
0	44 (97.78)	38 (84.44)	0.027
1	1 (2.22)	5 (11.11)	
2	0 (0.00)	2 (4.44)	
Motional discomfort (1 week)			0.617
0	37 (82.22)	35 (77.78)	
1	7 (15.56)	9 (20.00)	
2	1 (2.22)	0 (0.00)	
3	0 (0.00)	1 (2.22)	
Motional discomfort (1 month)			0.007
0	43 (95.56)	34 (75.56)	
1	2 (4.44)	9 (20.00)	
2	0 (0.00)	2 (4.44)	
Motional discomfort (6 month)			0.008
0	44 (97.78)	36 (80.00)	
1	1 (2.22)	8 (17.78)	
2	0 (0.00)	1 (2.22)	

## Discussion

To achieve surgical outcomes with ESSA comparable to those of OP, the key lies in rigorous preoperative patient assessment. If a PTC exhibits extrathyroidal invasion or is too large, caution is advised in selecting endoscopic surgery [19]. In addition, when performing operations under endoscopy with ultrasonic scalpels or other long-shaft instruments, ESSA is certainly less comfortable and meticulous than OP which uses short-handled instruments under direct vision. This is particularly evident when dealing with blood vessels at the upper pole of the thyroid gland and retracting the thyroid gland.

In this randomized clinical trial, we compared OP with ESSA in hemithyroidectomy and CND for PTC patients. A number of indicators including safety, effectiveness, cosmesis, and functional preservation of the anterior cervical region were fully assessed between the two groups. Our results indicate that there were no significant differences in general clinical data and complications between the two groups, but the operation duration of the ESSA group was significantly longer than that of the OP group (< 0.001); in terms of functional preservation of the anterior cervical region and cosmetic satisfaction, the ESSA group was significantly better than the OP group.

Unlike medullary thyroid carcinoma which has a relatively low incidence rate and poor prognosis [20], PTC has a favorable prognosis and increasing incidence rate among young people [1, 21, 22], leading to growing focus on cosmesis and functional preservation in thyroid surgery. In terms of functional preservation, in addition to the RLN [23] and parathyroid gland (PG) [24] which we have already been familiar with, functional preservation and cosmetic outcomes of the anterior cervical region are getting more and more attention [15, 25]. Although reliable, non-invasive and cost-effective transcutaneous laryngeal ultrasonography is currently available for evaluating preoperative vocal cord function, otolaryngologist-performed laryngoscopic vocal cord assessment remains a routine practice in our center [26]. For patients with preoperative vocal cord abnormalities on either the affected or contralateral side, open surgery rather than endoscopic surgery is recommended. In this study, no cases of recurrent laryngeal nerve (RLN) injury were observed in either the ESSA group or the OP group.

The mechanism of discomfort in the anterior cervical region is still unknown and needs to be examined. Current research suggests that discomfort in the anterior cervical region after surgery may be caused by the adhesions formed during thyroid surgery by the free trachea, esophagus and strap muscles, which fix each other [6, 15]. However, adhesions, destroying of the anterior cervical fat pad and separation of the anterior fascia of strap muscles are all inevitable occurrences during thyroid surgery, especially OP. In contrast, ESSA does not need to destroy those passes. It utilizes the natural gap between the SCM, without the need to break through the anterior fat pad of the neck and the anterior fascia of strap muscles. Only the posterior fascia of strap muscles needs to be freed, which is inevitable for thyroid resection. More dissociation and destruction mean more adhesions formed between the esophagus, strap muscles, skin, and trachea, which might result in more postoperative discomfort in the anterior cervical region.

Conventional OP leaves an exposed scar on the neck, which not only affects cosmesis, but may also affect the patient’s QoL to some extent [27]. However, ESSA can effectively solve this problem because the upper edge of the incision made in ESSA is located in the lower edge of the clavicle, so that most clothing can perfectly cover it. Besides, its cut length is 3.5 cm, which is shorter than the cut length of 4 cm in OP. In addition, due to its lateral approach, ESSA works better in exposing and protecting the RLN and PG than OP which provides a view from the center of the neck and makes it difficult to expose the RLN and PG located on the lateral side of the thyroid. On the other hand, compared to axillary approach, ESSA offers a shorter distance from the thyroid gland and requires lower technical requirements for laparoscopy.

As a gasless endoscopic surgery, ESSA can avoid many complications caused by CO2 [28]. However, also it still has some technical shortcomings, such as the difficulty in exposing the RLN at the entry point of the larynx due to obstruction of the trachea when removing the contralateral thyroid gland and the possibility to accidentally injure the supraclavicular nerve [29] and cause corresponding symptoms. In addition, according to our research results, ESSA usually requires longer operation duration than OP, mainly due to the preparation of endoscopic instruments and the establishment of endoscopic working spaces. Meanwhile, for experienced surgeons, this time gap is relatively reduced.

In our study, it was found that the patients in the ESSA group had fewer complaints about neck discomfort compared to those in the OP group at 1 and 6 months postoperatively, and that there was no difference between the two groups one week after surgery, probably because adhesions have not yet been formed in the first week after surgery.

The main outcome measure in this study was discomfort in the anterior cervical region, which was recorded and evaluated based on the patient’s subjective description. Although we used a scoring scale to get more accurate evaluation, it was still difficult to eliminate recording bias. In addition, the patients included in this study were from a single group in our center, with a small sample size. In order to gain more accurate experimental results and draw more representative research conclusions, subsequent studies need to obtain a larger-size sample from multiple centers. In addition, although studies have shown that endoscopic thyroid surgery yields favorable long-term prognostic outcomes [19], the follow-up period of this study is relatively short, which makes it impossible to accurately assess long-term effectiveness. Therefore, the extension of the follow-up period serves as a direction for future research. Besides, whether to routinely perform CND is still under investigation [30].

## Conclusions

The results of our research indicate that ESSA has the same safety and effectiveness as OP in unilateral thyroidectomy and CND, with better cosmetic outcomes and less discomfort in the anterior cervical region. ESSA may provide a better alternative for early unilateral PTC patients.

## Data Availability

The authors declare that all data presented in this manuscript are authentic, accurate, and have not been fabricated, falsified, or selectively reported.
